# Photovoice-Based Assessment of Weight Management Experiences of Breast Cancer Patients Treated with Tamoxifen

**DOI:** 10.3390/ijerph17124359

**Published:** 2020-06-18

**Authors:** Jung Suk Park, Jeong-Won Han, Jin Hyuk Choi, Kyoung Chun Lee

**Affiliations:** 1 College of Nursing, Kosin University, 262 Gamchen-ro (Jang Gi Ryeo-ro), Seo-gu, Busan 49267, Korea; cooler1978@kosin.ac.kr; 2 College of Nursing Science, Kyung Hee University, 26, Kyunghee-daero, Dongdaemun-gu, Seoul 02453, Korea; 3 Department of Breast Surgery, Kosin University Gospel Hospital, 262 Gamchen-ro (Jang Gi Ryeo-ro), Seo-gu, Busan 49267, Korea; drchoijinhyuk@gmail.com; 4 Department of Breast Surgery, Saegyero Hospital, 42, Jonghabundongjang-ro, Dongnae-gu, Busan 47876, Korea; gskoom@hanmail.net

**Keywords:** breast, neoplasm, body weight

## Abstract

In this study, an in-depth analysis of weight management experiences of breast cancer patients treated with tamoxifen is conducted, thereby providing basic data to help develop a multidimensional strategy to reduce recurrence and increase the survival rate of breast cancer patients. Study participants included nine breast cancer patients who were treated with tamoxifen at Kosin University Hospital and Saegyero Hospital in Busan Metropolitan City, Korea. This study employed the photovoice methodology. Participants described the need for family support and cooperation with weight management, provision of personalized weight management programs by medical institutions, provision of information on weight management programs by the community, and financial support for the weight management programs for breast cancer patients at the national level. This study emphasized the importance of weight management for breast cancer patients treated with tamoxifen and collected and analyzed vivid opinions of these patients using photos taken by them.

## 1. Introduction

The global average incidence rate of breast cancer stands at 11.9%, with a mortality rate of 6.4% and a 5-year survival rate of 87% [1]. In Korea, the breast cancer rate has been increasing by 4% annually from 2007 to 2015, with the 5-year survival rate reaching 92.3% [2]. Treatments for breast cancer vary widely and include surgery, chemotherapy, radiation therapy, and hormone therapy. Among these, hormone therapy is used as a supplementary treatment to prevent recurrence and remote metastasis after treatment for advanced breast cancer patients [3]. Tamoxifen, in particular, is a leading hormone drug for breast cancer patients and is used as the primary drug in hormone therapy when estrogen or progesterone receptors are positive. It has been reported to reduce the recurrence and metastasis of breast cancer [4,5]. In addition, the American Society of Clinical Oncology (ASCO) has revised its guidelines to state that tamoxifen is more effective in reducing recurrence and death rate if taken for an extended period of up to 10 years, rather than taking it for a short period [6]. However, if a breast cancer patient takes tamoxifen for an extended period, estrogen-like effects are observed in the endometrium. This in turn results in endometrial polyps, endometriosis, endometrial cancer, and decreased metabolic function that lead to weight gain due to increased body fat during treatment [7].

Although there are differences observed across countries and regions, studies from the United States and Japan have found that weight gain in breast cancer patients is related to cancer recurrence and affects survival rates [8,9,10]. Excess body weight at or after diagnosis is associated with a 20–43% higher risk of breast-cancer-specific and overall mortality, and twice the risk of breast cancer recurrence, compared to healthy-weight women [11]. Moreover, women diagnosed with breast cancer, in comparison to women without breast cancer, are more likely to develop cardiovascular diseases, which are strongly associated with overweight and obesity. Given the increased morbidity and mortality risk, maintenance of a healthy body weight or modest weight loss is strongly recommended in this population [12]. In particular, an increase in body mass index (BMI) in breast cancer patients is associated with an increase in body fat. Increased body fat increases the estrogen level in the blood, which in turn increases the risk of recurrence of breast cancer [13]. Additionally, a preliminary study on premenopausal women with breast cancer [14] found that those who were given tamoxifen had a constant increase in lipid profile and BMI. Another preliminary study on breast cancer patients who were given tamoxifen over the past five years [15] found that this population has a high incidence of non-alcoholic fatty liver disease. This suggests that they are at a high risk of an increase in BMI. Thus, it can be concluded that weight control is an important healthcare goal for breast cancer patients treated with tamoxifen [16].

Previous studies on weight management in breast cancer patients have shed light on the experience of health promoting behaviors [17], self-care support [18], and appearance management [19]. In foreign countries, studies were conducted on breast cancer patients in relation to their knowledge, experience and attitude on oncogenes [20], experience of acute skin toxicity after breast cancer radiation treatment [21], survival experience [22], and fatigue experience [23]. Most of the previous studies on breast cancer patients have been conducted on patients who received surgery, chemotherapy, and radiation therapy. However, there are limited studies on the weight management experience of breast cancer patients who had been treated with hormone therapy. Another limitation is that prior studies related to weight management among breast cancer patients were conducted only at the individual level.

While breast cancer patients can manage their weight through personal efforts in diet and physical activity [24], a multidimensional management at the individual, interpersonal, community and public policy levels is essential for weight management [25]. In particular, breast cancer patients receive hormone therapy at an acute stage of the disease, which is when support from not only individuals but also families, health care workers, and communities becomes critical [26]. Thus, weight management among breast cancer patients goes beyond individual efforts and is truly effective when various resources influencing their medical condition are identified and an approach is taken at a multidimensional level [25]. Weight management in breast cancer patients is directly related to cancer recurrence and survival rates, and a multitude of factors need to be considered from the socio-ecological perspective in addition to the individual’s perspective. Thus, an in-depth analysis of the factors affecting weight management in breast cancer patients is required.

In this respect, photovoice based on the participatory action research (PAR) methodology drives changes in individuals and communities through a discussion between participants and researchers on photographs taken by participants themselves [27] and takes a multidimensional approach to weight management among breast cancer patients. Unlike conventional research, which is highly dependent on professionals or researchers, photovoice allows research participants to actively engage in the research process along with the researchers, enabling an active exchange by sharing their general thoughts and experiences. Paulo Freire, an educator and philosopher, claimed that education and knowledge go beyond the teacher–student hierarchy to be built on collaborative work. Based on this theoretical background, photovoice emphasizes an equal participation among researchers and research participants [28]. In particular, photovoice provides participants with opportunities to communicate their life experiences through the visual images they present. This serves to enhance their capabilities in the process, while also simultaneously providing alternatives to facilitate change in participants. Prior studies that involved photovoice show that aboriginal identities and traditional beliefs are important factors in the health management experience of native women diagnosed with breast cancer [29]. Moreover, health management of women with breast cancer requires multidimensional support to address socioeconomic inequalities by race in addition to personal efforts [30]. Furthermore, studies on the quality of life of breast cancer survivors have also reported multiple approaches at different levels involving photovoice, such as exploring community support resources and providing role models, in addition to personal efforts [31]. Therefore, this study intends to provide basic data to help develop a weight management strategy for breast cancer patients through in-depth analyses of their experiences of weight management using photovoice after receiving tamoxifen in Korea.

## 2. Materials and Methods

### 2.1. Recruitment and Study Participants

The participants of this study were nine breast cancer patients who were treated with tamoxifen at Kosin University Hospital and Saegyero Hospital in Busan Metropolitan City, Korea. The age range of the participants was from 53 to 65 years. Specific criteria for selecting participants for the study were as follows: (1) those who had undergone surgery or chemotherapy or radiation therapy after diagnosis of breast cancer, (2) those who had been administered tamoxifen hormone medication for hormone therapy, (3) persons who, at the time of the study, did not have a recurrence or metastasis of breast cancer, (4) those who comprehended the purpose and method of research and who agreed to participate, (5) and those who were not administered psychiatric medications. This was a qualitative study; data collection and analysis were performed until the meaning and subjects of experience were saturated, using an objective sampling method according to the appropriateness and sufficiency criteria suggested by Morse and Field [32]. Particularly, the photovoice applied in this study is a form of participatory action study, rather than the random sampling of the traditional positivism studies. It focuses on revealing specific study topics by collecting groups and strengthening representativeness, mostly using a purposive sampling method [33]. Since the ideal number of participants for the photovoice studies is generally between seven to ten people [34], this study was conducted with nine participants.

### 2.2. Ethical Consideration

The selection process for the participants was as follows. This study recruited and selected the participants after acquiring permission from the Institutional Bioethics Advisory Board (KUGH 2018-11-025) of Kosin University. Following a breast surgeon’s identification of the health records of visiting outpatients, the purpose of the study was explained to the candidates meeting the selection criteria, who were then recruited. The participants were provided with sufficient information, namely an explanation of and counseling on the study, and then written consent was taken in an environment of free decision making. It was explained that if patients or carers do not wish to participate, they do not have to agree and that there is no disadvantage.

### 2.3. Measurement

#### 2.3.1. Training for Participants

Education for research participants was conducted in the seminar room at Saegyero Hospital on 3 June 2019. The researchers introduced the purpose and the content of the study as well as the method and the process of photovoice to the participants selected for this study. Moreover, the researchers educated them on how to write their own opinions on photography and photos. During training, handouts and detailed information regarding photography, participants, methods, research methods, processes, and research ethics was provided. There were four themes of photography available to the participants of this study. Since the participants of this study experienced the need for and importance of weight management while undergoing hormone therapy, the researchers organized questions into themes such as meaning-making of a person’s experience of weight management, factors they felt were promoting or hindering weight management in their lives, and strategies needed for weight control. These questions were as follows:

(1) What does weight management represent for me?
(2) What helps me manage my weight in my life?
(3) What comes in the way of weight management in my life?
(4) What do I need to do in my life to control my weight?

#### 2.3.2. Photo Shoots and Submission

Photos were taken and submitted during 6–19 June 2019. For about two weeks, the participants were asked to freely take pictures of their thoughts on each of the above subjects. They were then asked to select three photos for each subject that they felt were the most meaningful and representative of their thoughts, followed by an explanation. The photographs were sent to the researchers’ e-mail, which they printed to minimize inconvenience for the participants. The machines used by participants in this study for photography were cell phones, as it was determined in consultation with participants that cell phones were frequently used and participants were familiar with their operation.

#### 2.3.3. Primary Data Analysis

Primary data analysis was performed from 28 June to 8 July 2019. The researchers analyzed the photos received and participants’ opinions about these photos to derive the key themes. The materials discussed by the group were evaluated by checking for consistency through a cross-analysis among the researchers. The results were rearranged through discussion.

#### 2.3.4. Group Discussion by Theme

The group discussions were conducted in the seminar room at Saegyero Hospital on 9 July 2019. Based on the results of the primary data analysis, the researchers conducted a group discussion by subject to confirm whether the researchers analyzed the photos and the interpretation of the photos accurately and to increase the validity of the results of the study. Further comments from participants were also confirmed. The subject group discussion was conducted in accordance with the SHOWeD Photovoice Engagement Interview Techniques for photos taken by the study participants. Each participant described their own content and reasons, as well as their answers to the interview questions. In addition, the participants shared their experiences and opinions on the photographs taken by other participants and drew and discussed common themes. The questions in SHOWeD Photovoice Engagement Interview Techniques are as follows:

(1) What do you see here?
(2) What is really happening here?
(3) How does this relate to our lives?
(4) Why does this problem or this strength exist?
(5) What can we do about this?

Specific processes for the group discussion by topic were as follows. Participants discussed the submitted photos in two groups. In each group, there were two discussion meetings; each meeting was divided into two sessions for discussion by topic and comprehensive discussion. During these sessions, the researchers acted as hosts. In the discussion by topic, the discussion was conducted by dividing it into four topics: the meaning of weight management, the factors that help weight management, the factors that prevent weight management, and the necessity of weight control. The group discussion by topic required participants to select one of the pictures they had submitted that they thought was most representative of their thoughts on each topic. This was followed by comprehensive discussion. In addition, the researchers asked participants to discuss what they needed from their families, health care workers, communities, and countries for weight management from the perspective of breast cancer patients.

#### 2.3.5. Comprehensive Discussion

The comprehensive discussion was conducted in the seminar room at Saegyero Hospital on 9 July 2019. After the discussion by topic, participants were asked to assign a title for the photos selected and then pin them all on a large blackboard. This allowed participants to look at the photos on display together and to group and categorize the relevant factors by topic. On viewing the pictures divided according to topics, participants named each category of factors related to weight management among breast cancer patients until they arrived at a consensus. Further questions asked by researchers about “their requirements from families, health care workers, communities and countries for weight management from their perspective as breast cancer patients” required participants to write a memo to the group and categorize relevant factors.

#### 2.3.6. Secondary Data Analysis

The secondary data analysis was conducted from 10 to 30 July 2019. After closing the general discussion, researchers organized the recorded parts of the discussion, and memos were rearranged to organize participants’ viewpoints vividly in an orderly manner. This was followed by a comprehensive analysis of photos submitted and their details, along with corrections, additions, or complements made during the group discussion. The data analysis in this study was conducted in accordance with steps suggested by Smith et al. [35]. In the first step, the researchers intended to obtain the understanding of and insight into participants’ experience in the research phenomenon while repeatedly reading copied text data. Then, in order to topicalize text data, they analyzed the manuscripts of the first individual case by sentence and paragraph breaks and recorded researchers’ exploratory comments. An exploratory comment refers to a researcher underlining seemingly important text while reading manuscripts and recording their observations on it. For the next step, the researcher recorded an emergent theme through an initial analysis of the exploratory comments after a rereading of the manuscripts. The next step involved categorizing similar themes to create a larger theme, followed by a cross-case analysis to uncover relevance and patterns among these themes. The final step of analysis involved reflecting on prominent common attributes and participants’ unique individual characteristics to compare similarities and differences among cases before drawing a subordinate theme.

#### 2.3.7. Credibility and Validity in Study

This study was intended to secure credibility and validity in research by fully considering truth value, applicability, consistency, and neutrality in compliance with evaluation criteria for strictness within qualitative research suggested by Guba and Lincoln [36]. To identify whether the first photovoice analysis accurately reflected meanings and perspectives intended by research participants, researchers secured truth value by means of a cross-analysis between researchers and by reidentifying an ambiguous definition through one-on-one interviews with the participants. On top of that, they selected participants suited for the purpose of the research, drew conclusions in an orderly manner according to the thematic analysis process by Smith et al. [35] to keep research consistent, and strived to help draw consistent conclusions through the audit process. The audit process was directed by a nursing professor holding many years of experience in qualitative research. Finally, researchers strived to increase objectivity by meeting criteria for truth value, applicability, and consistency [37] and to secure neutrality by accepting participants’ as-is experience without influencing or manipulating situations intentionally.

## 3. Results

### 3.1. General Characteristics of Participants

The participants of this study consisted of nine breast cancer patients. The age of participants ranged from 53 to 65 years, with four participants in their 50s and five in their 60s. The participants’ cancer stages ranged from stage 1 to stage 3, with three at stage 1, four at stage 2, and two at stage 3; eight participants underwent a total resection and one underwent a subtotal resection. Four of the participants received both chemotherapy and radiation therapy, while five received only chemotherapy. The tamoxifen administration period ranged from five to eight years. The increased weight range after tamoxifen administration in the participants was 2–10 kg, with an average increase of 4.8 kg (Table 1).

### 3.2. Photovoice Outcomes

**Figure 1:**
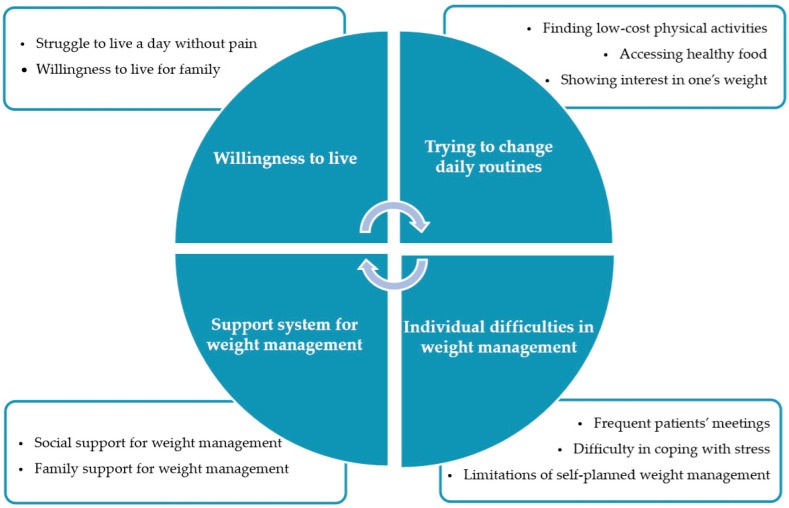
Theme of weight management experiences of breast cancer patients treated with tamoxifen.

The analysis of participants’ photovoice photos, explanatory notes, and group discussions revealed four emergent themes and ten subordinate themes. It turned out that weight management in breast cancer patients stems from the willingness to live with their families without pain. They also looked for physical activities at low cost for weight management and eating healthy food, and showing an interest in weight was indicative of promoting weight management. The finding also revealed that weight management was interrupted by factors such as attending frequent patients’ meetings, lack of ways to cope with stress, and managing weight control on their own. Breast cancer patients demanded that social assistance and family support should be arranged for them to maintain weight effectively. Further details on these themes can be found in Table 2 and Figure 1

#### 3.2.1. Superordinate Theme 1: Willingness to Live

(1) Struggle to live a day without pain

Breast cancer patients said they are physically and emotionally exhausted during treatment following the cancer diagnosis, such as surgery, chemotherapy, and hormone therapy. They also said they are living each day in emotional distress and fear of cancer recurrence. The patients had heard from the medical team about the importance of weight management from the time they took tamoxifen. They acknowledged that weight management now depends on their efforts to live a day without pain while expecting to be free from cancer.

*“To me, weight management means to live a healthy life even if I just have one day left to live. My goal is to live a healthy, pain-free life for at least a day rather than living long.”*

(Participant 1)

(2) Willingness to live for family

Breast cancer is the most commonly occurring cancer in women, and all participants are playing the roles of mothers at their homes. They expressed their desire to live for their family as a mother or as a wife. The participants expressed their feelings; they said that they still have a lot of things to do for their children and should, therefore, stay healthy for their sake. They also said that successful weight management is related to their strong willingness to spend as much time with their families as possible.

**Figure 2:**
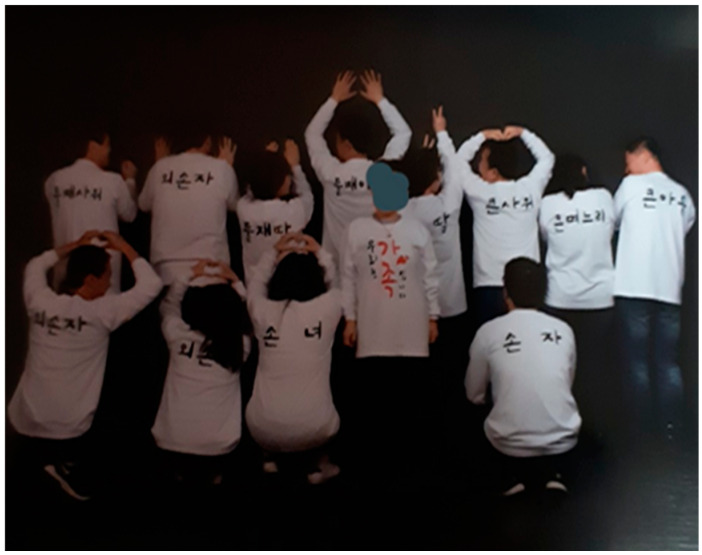
Meaning of weight management for breast cancer patients.

*“Family is a goal in my life and I believe my family will be happy with me. If cancer comes back, putting me back in the hospital, it will certainly make my family sad more than anyone else. This is why I am determined to keep my weight under control for the sake of my family.”*

(Participant 9) (Figure 2)

#### 3.2.2. Superordinate Theme 2: Trying to Change Daily Routine

(1) Finding low-cost physical activities

Participants complained about physical fatigue due to their disease and repeated treatment and wanted to rest at home despite showing concern about weight gain caused by tamoxifen; they sought alternate ways for weight management. As long-term cancer treatment was causing an economic burden for them, patients were going out of their way to seek and practice low-cost physical activities in their daily life among the many methods of weight management. The research participants expressed their highest preference for attending free workout programs (e.g., yoga, Pilates, and aerobics) held in public healthcare centers.

*“I started to go hiking for weight management. Going hiking is free of charge. I love the mountains, and once I get there, the clean mountain air refreshes me as if my pain instantly disappeared.”*

(Participant 3)

(2) Accessing healthy food

Research participants said they relied largely on ready-to-eat food options for each meal because they spent most of the time at home, alone, with reduced appetite, even before they were diagnosed with cancer. They also added that dinner menu items were driven by their family members’ choice rather than theirs. They mentioned most of the food intake at home was high in calories, making it difficult for them to maintain proper weight. They added the cancer pushed them towards making efforts to gradually change just their own menus to help their weight management.

**Figure 3:**
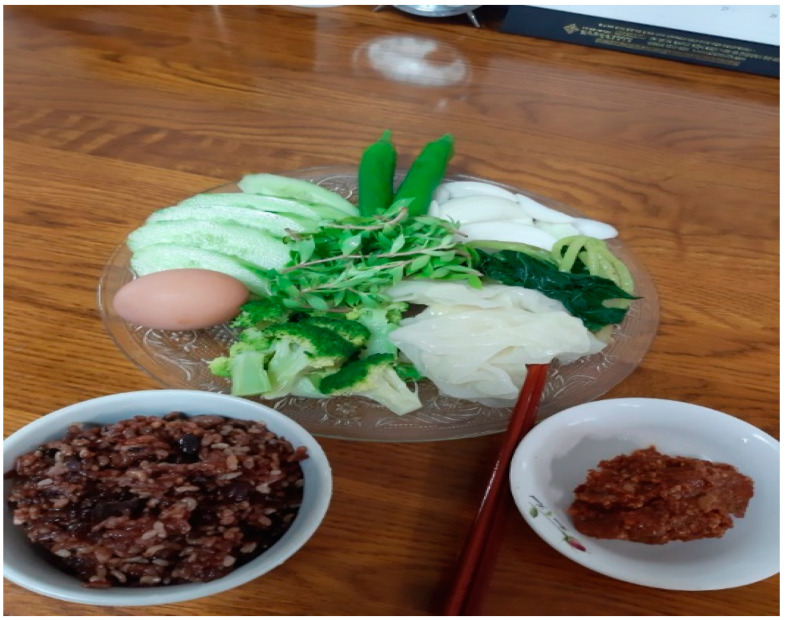
Weight management facilitators for breast cancer patients.

*“Diet menus are of great help in maintaining my weight. Before I fell sick, my favorites were carbohydrate-rich foods, fried foods, and sweet stuff. I, probably, got breast cancer because of my dietary habits. So, I mainly blanch fresh seasonal vegetables before eating for my weight management.”*

(Participant 4) (Figure 3)

(3) Showing interest in one’s weight

Participants said they normally have little interest in their weight. Now that most of them were close to menopause, they took weight gain as natural; they said none of them weighed themselves even out of curiosity during the day. However, upon hearing from a doctor about the dangers of weight gain when taking tamoxifen, they said they developed a habit of weighing themselves. It was mentioned that they weighed themselves either in the morning or evening to set up plans for weight management for the following day, and they reserved time for verifying the effect of their methods.

*“The scale helps me maintain proper weight. I weigh myself once a day. While weighing myself, I also check the daily amount of food eaten or amount of exercise, along with plans for tomorrow’s weight management.”*

(Participant 5)

#### 3.2.3. Superordinate Theme 3: Individual Difficulties in Weight Management

(1) Frequent patients’ meetings

Among the participants, patients’ meetings work as the only social support system for sharing similar experiences with one another. The meeting became a critical support system because it is a platform where they share necessary information and activities with one another. However, as they started to take tamoxifen, they said the patients’ meetings led to an actual setback in their weight management. The patients’ meetings are held at least once a month and in many cases once a week. They said they find themselves eating a lot of food and refreshments at the meeting and fail to meet their weight management goals. They added that, on the day of the meeting, they witnessed weight gain due to overeating, which would certainly take a lot of time and effort to return to their normal weight. They described this as their dilemma over whether to attend the patients’ meetings or not.

*“I have four or five patients’ meetings to attend each month. I eat a lot there. It makes perfect sense in theory that I avoid overeating and achieve weight loss until I go there, but the problem is that I cannot help attending the meetings.”*

(Participant 8)

(2) Difficulty in coping with stress

While taking tamoxifen to maintain everyday life, research participants were given more opportunities to meet their family than when they were hospitalized. Most of all, the increased opportunities to encounter their in-laws allowed them to experience a conflict with in-laws which any other daughter-in-law in Korean society faces. Such a conflict is said to work to induce a high level of stress among the participants. Being cancer patients, they have some restrictions, such as those on drinking alcohol or playing rough sports—activities that are otherwise regarded as a normal means of easing stress. They said this made them explore other ways of easing stress, such as eating delicious food, well known to relieve stress, and chatting with peers in patients’ meetings. In spite of the awareness that stress relief by eating excess food or comfort food hardly works in weight management, research participants said that, as cancer patients, they find it difficult to discover alternate ways to cope with stress.

*“It is the stress from my in-laws that prevents me from getting my weight under control. I turn to binge eating when stressed out.”*

(Participant 6)

(3) Limitations of self-planned weight management

Research participants were at home, alone in planning and dealing with weight management. As they frequently heard from the hospitals about the importance of weight management, they said they had to deal with the difficulty of making plans and managing them all by themselves because the hospital does not offer actual weight management plans. In addition to this, even when they stay at home, they make plans for exercise and diet for weight management without their family’s help. They said this lowered their willingness to manage weight, leading to failure in weight control in many cases.

**Figure 4:**
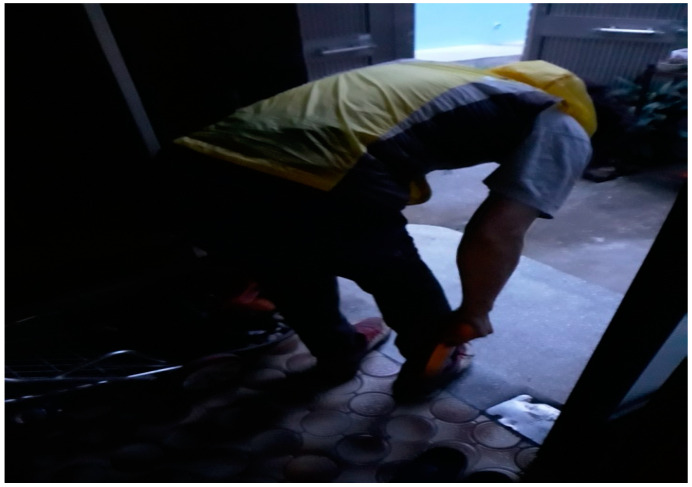
Weight management deterrents for breast cancer patients.

*“I am the only one in the family who works on weight control. To control my weight, I have to get up early in the morning or should exercise in a planned time segment of my daily routine. I am rather weak-willed and lazy, and I hardly go hiking, so I want to maintain weight by drinking detox juices and just keep burning fat.”*

(Participant 2) (Figure 4)

#### 3.2.4. Superordinate Theme 4: Support System for Weight Management

(1) Social support for weight management

Research participants expressed their thoughts about incurring heavy expenses in weight management as a burden, adding that there is a need for finding effective ways at low cost or with financial support enabling them to access various programs provided by communities or the government. Breast cancer patients refer to hiking as a physical activity for weight management, but in reality they say hiking costs the least in weight management. Though hiking was not a reasonable option for their weight management, research participants expressed that high-cost weight control options are likely to put a financial strain on their family; therefore, they thought hiking was the best option available to them. They said it would be helpful for their weight management if communities or governments provide financial support or a voucher system for breast cancer patients. They hoped that hospitals would also offer weight management programs for breast cancer patients instead of just emphasizing its importance. Furthermore, they also highlighted that the hospitals should advise on exercise regime and weight management programs that are tailored for individuals, rather than a uniform program. In addition, institutions need to provide easy-to-access programs regarding various physical activities being run in local communities.

**Figure 5:**
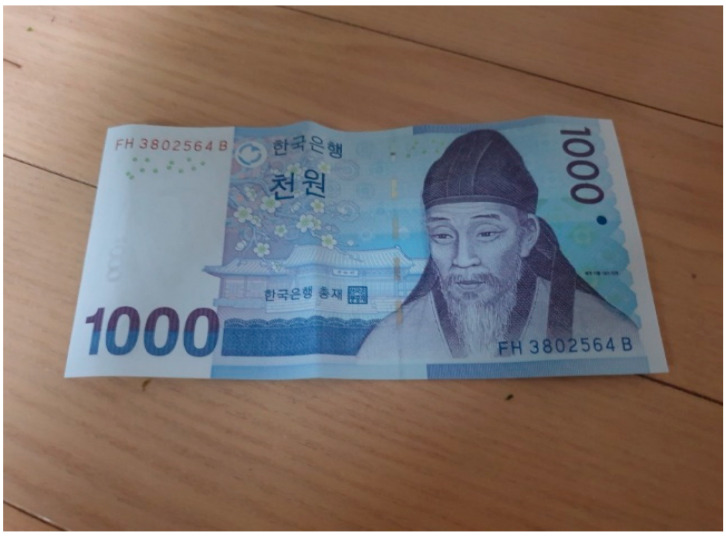
Support needed for weight management for breast cancer patients.

*“Treating breast cancer patients costs a lot, so many of them turn to hiking for financial reasons and give up other exercises. If the government builds sufficient voucher systems for exercise purposes, it will work out perfectly for us in terms of weight management. We can work out closer to home instead of going up the mountains.”*

(Participant 1) (Figure 5)

*“The hospital recommends that breast cancer patients should take part in laughter therapy, but its effects are rather insignificant among the participants. Everyone has their own lifestyle.”*

(Participant 3)

*“Energy exercises worked just great for me in managing my weight. But, there is no detailed information about which public healthcare center provides this program. I wish it was made easily accessible.”*

(Participant 5)

(2) Family support for weight management

Research participants addressed difficulties in weight management practiced in their own at home, expressing the need for weight management with their family’s help. Breast cancer patients, other than patients’ meetings, spend most of their time with their families. However, with none of the family members participating with interest in weight management, they found it most difficult to control their diet during family mealtimes. They suggested that it would be effective for breast cancer patients and their family members receive training and education from the hospital regarding weight management, because then they can get help through various channels for themselves and their families.

*“It appears that weight management is not something I can achieve on my own, and I would like to receive help from my family to make it work.”*

(Participant 5)

## 4. Discussion

The purpose of this study was to provide basic data to help develop strategies to reduce recurrence rates and increase survival rate among breast cancer patients by analyzing the experiences of weight management among these patients in an in-depth manner, using photovoice.

First, in this study, the meaning of weight management for breast cancer patients was in having a healthy life as well as in an effort made by their respective families to this effect. These findings are similar to those presented in a study of breast cancer patients in the UK, which suggested that weight control for breast cancer patients meant living a healthy life with their families [38]. Breast cancer typically afflicts women; leading a healthy life through weight management in breast cancer patients implies recognizing a sense of responsibility, as it is the mother who cares for the whole family [39]. In addition, long-term breast cancer patients struggle to fulfill their multiple roles, and hence the desire to be healthier for their families and to care for them is an important driving force that supports their will to live [40]. Thus, caregivers who take care of breast cancer patients should have a more family-level approach to and understanding of the meaning of weight management for breast cancer patients. This will thereby help the patients strengthen their resolve for weight management.

Second, in this study, weight management promotion factors in breast cancer patients were exercise (especially muscular exercise), diet control, and weight management habits. These findings are similar to those presented by the Guidelines for Breast Cancer Patients in the United States [41]. These guidelines note that breast cancer patients should prevent obesity, develop a habit of regular exercise and consumption of fruits and vegetables, and limit high-calorie foods in order to reduce recurrence and improve survival rate. In addition, the National Comprehensive Cancer Network (NCCN) guidelines suggest that keeping a healthy body weight, regular physical activity, healthy diets, minimizing alcohol intake, avoiding exposure to UV rays, and regular checkups are essential for leading a healthy life for cancer patients [42]. Judging from these results, participants in this study were aware of all these measures through experience and put them in practice. In addition, this study found that breast cancer patients measured their weight daily, and this helped them with weight control. A similar finding was noted among Finnish adults who had relatively high weight loss and retention rates when they measured their weight daily [43]. This study confirmed that weight management does not happen in a short period of time, and long-term efforts through self-regulation are needed. Moreover, it is important for care workers and communities to provide self-regulating programs for weight control of breast cancer patients.

Third, weight management deterrents among breast cancer patients were participation in patient reunions, stress, and lack of commitment to weight control. Breast cancer patients, who have experienced shrinking human relationships because of their disease, value a patient reunion because it allows them to experience deeper human connections and provides them with a space to share their experience with their disease [44]. In addition, breast cancer patients actively participate in social activities such as hobbies and volunteering activities through self-help groups, thus expanding their social circle [40]. However, for breast cancer patients, self-help groups can be perceived as insufficient social support for weight management or as an interruption [45]. Furthermore, stress and lack of commitment to weight control were also identified as deterrents to weight management [46]. Thus, self-help groups of breast cancer patients need experts to provide accurate information and management systems for future care. In addition, stress management and motivational programs for breast cancer patients should be facilitated by medical professionals and should incorporate a social perspective to weight management.

Fourth, this study demonstrated that the support required for weight management by breast cancer patients was that of their family’s interest and cooperation in weight management, the provision of personalized weight management programs by medical institutions, the provision of information on weight management programs by the community, and state-sponsored weight management or a state subsidy for weight management. This finding echoes a previous study on African Americans which found that the provision of programs at various levels, such as enhancing self-efficiency in losing weight with breast cancer patients’ weight loss programs, social support, and providing dietary and exercise control programs in the community, are effective in breast cancer patients’ weight loss and maintenance [47]. In addition, studies on exercise for breast cancer patients show that they prefer exercising under supervision, exercising with buddies, tailored exercise programs, and fun exercises. Given that the programs that breast cancer patients want in this study are already structured in prior studies, overseas, and provided in the community, weight management programs for this population need a multidimensional approach that goes beyond the individual level. Moreover, it is especially important that health care providers organize systematic training and programs on weight management and connect them to the community system. In addition, breast cancer patients also need social efforts, including establishment of certain systems, to be provided for them, as the lack of information on program details, such as dates and venue, restrains the patients from accessing suitable programs. It is also necessary to establish a system to make weight management easier and more comfortable for breast cancer patients through voucher schemes for such programs, as suggested by participants in the study, given that these programs often put financial pressure on patients and many choose to go hiking instead.

## 5. Conclusions

This study was conducted to review weight management experiences and find alternative methods for breast cancer patients who were treated with tamoxifen in Korea by using photovoice as a research methodology. As a result of this study, participants described the need for family support and cooperation in weight management, provision of personalized weight management programs by medical institutions, provision of information on these programs by the community, and financial support for these programs for breast cancer patients at a national level. Based on the results of this study, it is important for health care professionals caring for the breast cancer patients treated with tamoxifen to educate the patients about the meaning and importance of weight management at the beginning of the treatment and to link the weight management programs in hospitals and communities. Additionally, since the results of this study revealed that breast cancer patients wanted to manage their weight with the help of their families, it is critical to seek ways to include their families in these programs. Professional management, such as stress relievers and diet control for the self-help groups of these patients, should be provided by including trained nurses in these groups. At the community level, it is necessary for private organizations, health centers, and universities to develop weight management programs for these patients in order to deliver information continuously and to connect them with hospitals. At the national level, it is recommended to provide financial support for developing and maintaining weight management programs for breast cancer patients. This study is different from previous studies as it emphasizes the importance of weight management for breast cancer patients treated with tamoxifen, and it collected and analyzed vivid opinions of the patients by utilizing photos that they had taken. However, since this study consists only of breast cancer patients injected with tamoxifen, it is limited in its identification of the meaning of weight management for patients undergoing other hormone treatments. Based on the results of this study, development and application of weight management programs for breast cancer patients is needed.

## Figures and Tables

**Table 1:** General characteristics of participants.

ID	Age	Marital Status	Stage of Disease	Type of Operation	Chemotherapy	Radiation Therapy	Duration of Tamoxifen Therapy	Weight Gain after Tamoxifen Therapy	Weight Gain Degree (kg)
1	54	Married	1	Total mastectomy	Yes	No	5	Yes	2
2	53	Married	2	Total mastectomy	Yes	No	8	Yes	2
3	60	Married	3	Total mastectomy	Yes	Yes	5	Yes	4
4	62	Married	2	Total mastectomy	Yes	Yes	7	Yes	10
5	55	Married	2	Total mastectomy	Yes	No	5	Yes	10
6	57	Married	2	Total mastectomy	Yes	No	6	Yes	2
7	65	Married	1	Total mastectomy	Yes	No	5	Yes	2
8	64	Married	1	Subtotal mastectomy	Yes	Yes	5	Yes	7
9	62	Married	3	Total mastectomy	Yes	Yes	7	Yes	4

**Table 2:** Superordinate and subordinate themes of weight management experiences of breast cancer patients treated with tamoxifen.

Superordinate Themes	Subordinate Themes
Willingness to live	Struggle to live a day without pain
	Willingness to live for family
Trying to change daily routine	Finding low-cost physical activities
	Accessing healthy food
	Showing interest in one’s weight
Individual difficulties in weight management	Frequent patients’ meetings
	Difficulty in coping with stress
	Limitations of self-planned weight management
Support system for weight management	Social support for weight management
	Family support for weight management
